# Development of EMab-51, a Sensitive and Specific Anti-Epidermal Growth Factor Receptor Monoclonal Antibody in Flow Cytometry, Western Blot, and Immunohistochemistry

**DOI:** 10.1089/mab.2017.0028

**Published:** 2017-10-01

**Authors:** Shunsuke Itai, Mika K. Kaneko, Yuki Fujii, Shinji Yamada, Takuro Nakamura, Miyuki Yanaka, Noriko Saidoh, Saori Handa, Yao-Wen Chang, Hiroyoshi Suzuki, Hiroyuki Harada, Yukinari Kato

**Affiliations:** ^1^ Department of Antibody Drug Development, Tohoku University Graduate School of Medicine, Miyagi, Japan.; ^2^ Department of Oral and Maxillofacial Surgery, Graduate School of Medical and Dental Sciences, Tokyo Medical and Dental University, Tokyo, Japan.; ^3^ Department of Regional Innovation, Tohoku University Graduate School of Medicine, Miyagi, Japan.; ^4^ Department of Pathology and Laboratory Medicine, Sendai Medical Center, Miyagi, Japan.; ^5^ New Industry Creation Hatchery Center, Tohoku University, Miyagi, Japan.

**Keywords:** EGFR, monoclonal antibody, immunohistochemistry, oral cancer

## Abstract

The epidermal growth factor receptor (EGFR) is a member of the human epidermal growth factor receptor (HER) family of receptor tyrosine kinases and is involved in cell growth and differentiation. EGFR homodimers or heterodimers with other HER members, such as HER2 and HER3, activate downstream signaling cascades in many cancers. In this study, we developed novel anti-EGFR monoclonal antibodies (mAbs) and characterized their efficacy in flow cytometry, Western blot, and immunohistochemical analyses. First, we expressed the full-length or ectodomain of EGFR in LN229 glioblastoma cells and then immunized mice with LN229/EGFR or ectodomain of EGFR, and performed the first screening using enzyme-linked immunosorbent assays. Subsequently, we selected mAbs according to their efficacy in flow cytometry (second screening), Western blot (third screening), and immunohistochemical (fourth screening) analyses. Among 100 mAbs, only one clone EMab-51 (IgG_1_, kappa) reacted with EGFR in Western blot analysis. Finally, immunohistochemical analyses with EMab-51 showed sensitive and specific reactions against oral cancer cells, warranting the use of EMab-51 to detect EGFR in pathological analyses of EGFR-expressing cancers.

## Introduction

The epidermal growth factor receptor (EGFR) is a transmembrane receptor that is involved in cell growth and differentiation.^(1–3)^ EGFR is a member of the human epidermal growth factor receptor (HER) family of receptor tyrosine kinases. EGFR homodimers or heterodimers with other HER members, such as HER2 and HER3, activate downstream signaling cascades and control many biological processes. These pathways are frequently dysregulated via overexpression of EGFR in many cancers, including brain tumors, head and neck cancers, lung cancers, breast cancers, pancreatic cancers, kidney cancers, prostate cancers, ovary cancers, bladder cancers, and colorectal cancers.^(4)^

EGFR is the first receptor target against which monoclonal antibodies (mAbs), including cetuximab (a mouse-human chimeric mAb; IgG_1_) against colorectal cancers and head and neck cancers, panitumumab (a fully human mAb; IgG_2_) against colorectal cancers, and necitumumab (a fully human mAb; IgG_1_) against nonsmall cell lung cancers, have been developed for cancer treatment.^(5–7)^ Anti-EGFR mAbs possess several functional mechanisms, including antibody-dependent cellular cytotoxicity (ADCC), complement-dependent cytotoxicity (CDC), blocking dimerization or ligand binding, and EGFR endocytosis.

We have produced antipodoplanin (PDPN) cancer-specific mAbs (CasMabs), clone LpMab-2^(8,9)^ and LpMab-23,^(10,11)^ which specifically recognize cancer-type PDPN in tumor tissues. For this technology, it is critical that immunogens are produced using cancer cell lines, such as LN229 glioblastoma cells, which express cancer-specific glycan-attached membrane proteins. This method could be used to develop useful mAbs against multiple membrane proteins. In this study, we produced sensitive and specific mAbs against EGFR using this technology.

## Materials and Methods

### Cell lines

LN229, A431, Chinese hamster ovary (CHO)-K1, HEK-293T, MCF-10A, Met-5A, and P3U1 cell lines were obtained from the American Type Culture Collection (ATCC, Manassas, VA). HSC-2 and HSC-3 were obtained from the Japanese Collection of Research Bioresources (JCRB) Cell Bank (Osaka, Japan). LN229/EGFR and CHO/EGFR were produced by transfecting pCAG/PA-EGFR-RAP-MAP^(12)^ into LN229 and CHO-K1 cells using Neon transfection system (Thermo Fisher Scientific, Inc., Waltham, MA) and Lipofectamine LTX (Thermo Fisher Scientific, Inc.), respectively. A few days after transfection, PA tag-positive cells^(13)^ were sorted using a cell sorter (SH800; Sony Corp., Tokyo, Japan).

CHO-K1, CHO/EGFR, and P3U1 cell lines were cultured in RPMI 1640 medium (Nacalai Tesque, Inc., Kyoto, Japan), and LN229, LN229/EGFR, A431, HSC-2, HSC-3, HEK-293T, MCF-10A, and Met-5A cell lines were cultured in Dulbecco's modified Eagle's medium (Nacalai Tesque, Inc.), supplemented with 10% heat-inactivated fetal bovine serum (Thermo Fisher Scientific, Inc.), 100 units/mL of penicillin, 100 μg/mL of streptomycin, and 25 μg/mL of amphotericin B (Nacalai Tesque, Inc.) at 37°C in a humidified atmosphere containing 5% CO_2_ and 95% air.

### Animals

Female 4-week-old BALB/c mice were purchased from CLEA Japan (Tokyo, Japan). Animals were housed under specific pathogen-free conditions. The Animal Care and Use Committee of Tohoku University approved all the animal experiments described in this study.

### Hybridoma production

The ectodomain of EGFR with N-terminal PA tag^(13)^ and C-terminal RAP tag^(14)^-MAP tag^(12)^ (EGFRec) was purified from supernatant of LN229/EGFRec using anti-RAP tag as described previously.^(14)^ BALB/c mice were immunized using intraperitoneal (i.p.) injections of LN229/EGFR cells or 100 μg of EGFRec together with Imject Alum (Thermo Fisher Scientific, Inc.). After several additional immunizations, a booster injection of LN229/EGFR cells or 100 μg of EGFRec was intraperitoneally administered 2 days before harvesting spleen cells. Spleen cells were then fused with P3U1 cells using PEG1500 (Roche Diagnostics, Indianapolis, IN) or GenomONE-CF (Ishihara Sangyo Kaisha, Ltd., Osaka, Japan).

The resulting hybridomas were grown in RPMI medium supplemented with hypoxanthine, aminopterin, and thymidine selection medium supplement (Thermo Fisher Scientific, Inc.). Culture supernatants were screened using enzyme-linked immunosorbent assay (ELISA) with EGFRec, and mAbs were selected using flow cytometry (second screening), Western blot (third screening), and immunohistochemical analyses (fourth screening). MAbs were purified from supernatants of hybridomas cultured in Hybridoma-SFM medium (Thermo Fisher Scientific, Inc.) using Protein G Sepharose 4 Fast Flow (GE Healthcare UK Ltd., Buckinghamshire, England).

### Flow cytometry

Cells were harvested by brief exposure to 0.25% trypsin/1-mM ethylenediaminetetraacetic acid (EDTA) (Nacalai Tesque, Inc.). After washing with 0.1% bovine serum albumin (BSA)/phosphate-buffered saline (PBS), the cells were treated with 1 μg/mL of anti-EGFR (clone EMab-51) for 30 minutes at 4°C and then with Alexa Fluor 488-conjugated anti-mouse IgG (1:1000; Cell Signaling Technology, Inc., Danvers, MA). Fluorescence data were collected using EC800 or SA3800 Cell Analyzers (Sony Corp.).

### Western blot analysis

Cell lysates (10 μg) were boiled in sodium dodecyl sulfate (SDS) sample buffer (Nacalai Tesque, Inc.) and proteins were then electrophoresed on 5%–20% polyacrylamide gels (Wako Pure Chemical Industries Ltd., Osaka, Japan) and were transferred onto polyvinylidene difluoride (PVDF) membranes (Merck KGaA, Darmstadt, Germany). After blocking with 4% skim milk (Nacalai Tesque, Inc.), membranes were incubated with 10 μg/mL of anti-EGFR (clone EMab-51) and 1 μg/mL of anti-β-actin (clone AC-15; Sigma-Aldrich Corp., St. Louis, MO), then with peroxidase-conjugated anti-mouse IgG (1:1000 diluted; Agilent Technologies, Inc., Santa Clara, CA), and were finally developed using ImmunoStar LD (Wako Pure Chemical Industries Ltd.) using a Sayaca-Imager (DRC Co. Ltd., Tokyo, Japan).

### Determination of the binding affinity using flow cytometry

LN229 and A431 (2 × 10^5^ cells) were suspended in 100 μL of serially diluted mAbs (6 ng/mL–50 μg/mL), and Alexa Fluor 488-conjugated anti-mouse IgG (1:200; Cell Signaling Technology, Inc.) was then added. Fluorescence data were collected using a cell analyzer (EC800; Sony Corp.). The dissociation constants (*K*_D_) were calculated by fitting the binding isotherms using the built-in one-site binding models in GraphPad PRISM 6 (GraphPad software, Inc., La Jolla, CA).

### Immunohistochemical analyses

Oral cancer tissues were purchased from US Biomax, Inc. (Rockville, MD). Histologic sections of 4-μm thickness were deparaffinized in xylene and were then rehydrated and autoclaved in EnVision FLEX Target Retrieval Solution High pH (Agilent Technologies, Inc.) for 20 minutes. Sections were then incubated with 5 μg/mL of EMab-51 for 1 hour at room temperature and were then treated using an EnVision+ Kit (Agilent Technologies, Inc.) for 30 minutes. Color was developed using 3,3-diaminobenzidine tetrahydrochloride (Agilent Technologies, Inc.) for 2 minutes, and sections were then counterstained with hematoxylin (Wako Pure Chemical Industries Ltd.). The intensity of staining was evaluated as —, 1+, 2+, 3+.

## Results

### Production of anti-EGFR mAbs

In this study, we immunized mice with LN229/EGFR or purified recombinant EGFRec from culture supernatants of LN229/EGFRec cells. A booster i.p. injection of LN229/EGFR or EGFRec was also administered, and culture supernatants were then screened for binding to purified EGFRec using ELISA. As a second screening, we used flow cytometry to assess reactions with LN229 and LN229/EGFR cells. LN229 cells express endogenous EGFR^(15)^; therefore, a stronger reaction against LN229/EGFR was required.

A total of 38 clones were generated from immunizations with LN229/EGFR, and 62 were generated from immunizations with purified EGFRec. Of 100 mAbs, 94 clones reacted with A431 cells, which express endogenous EGFR.^(16)^ Subsequently, mAbs were further selected according to their efficacy on Western blot analysis. These analyses identified only one clone EMab-51 (IgG_1_, kappa) from EGFRec immunizations that were useful for Western blot analysis.

### Characterization of EMab-51

**FIG. 1.**
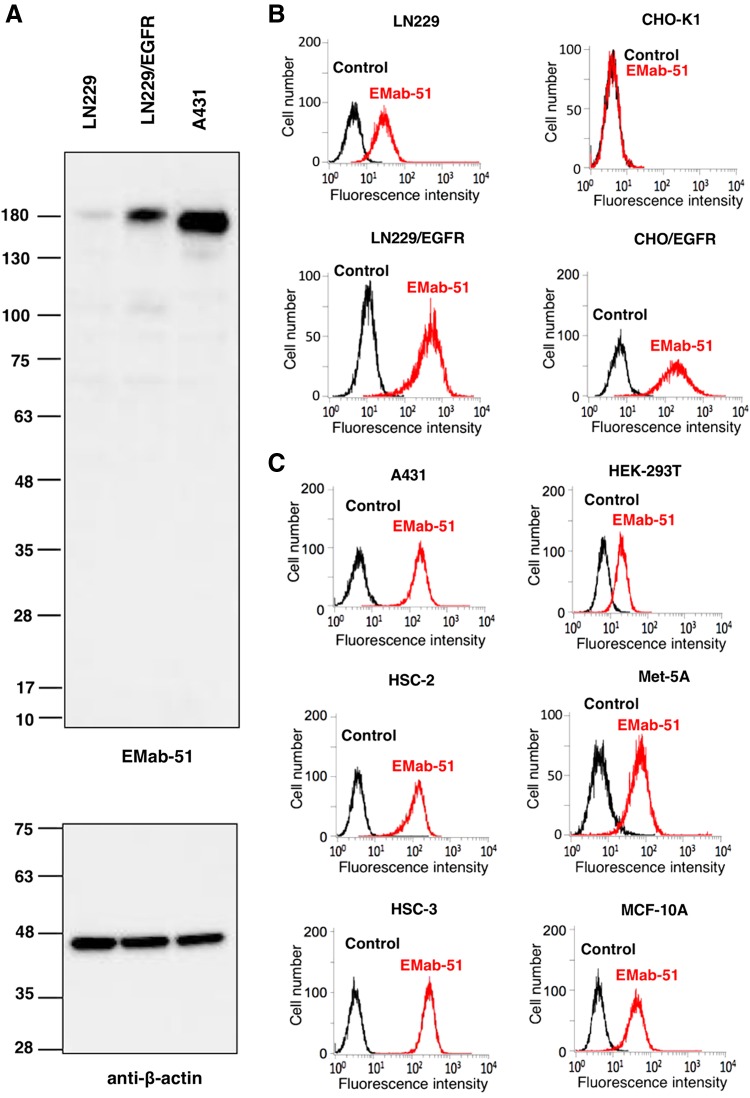
Characterization of EMab-51. **(A)** Western blot using EMab-51; cell lysates (10 μg) were electrophoresed and proteins were transferred onto polyvinylidene difluoride (PVDF) membranes. After blocking, membranes were incubated with 10 μg/mL of EMab-51 or 1 μg/mL of anti-β-actin (AC-15) and then incubated with peroxidase-conjugated anti-mouse IgG. **(B, C)** Flow cytometry with EMab-51; cells were treated with 1 μg/mL of EMab-51 followed by Alexa Fluor 488-conjugated anti-mouse IgG; black line, negative control.

On Western blot analyses against LN229,^(15)^ LN229/EGFR,^(12)^ and A431 cells,^(16)^ EMab-51 detected ∼180-kDa strong signal in LN229/EGFR and A431 cells, and the weak signal in LN229, indicating that EMab-51 is very useful for Western blot analyses (Fig. 1A). Subsequent flow cytometry demonstrated that EMab-51 reacted with LN229/EGFR more strongly than with endogenous EGFR-expressing LN229 brain tumor cells (Fig. 1B). EMab-51 also reacted with CHO/EGFR and did not react with the parental cell CHO-K1, indicating that EMab-51 is specific for EGFR (Fig. 1B). EMab-51 also recognized endogenous EGFR in A431 epidermoid carcinoma cells,^(16)^ HSC-2 and HSC-3 oral squamous cell carcinomas,^(11)^ HEK-293T renal epithelial cells,^(17)^ Met-5A mesothelial cells,^(18)^ and MCF-10A breast epithelial cells^(19)^ (Fig. 1C).

**FIG. 2.**
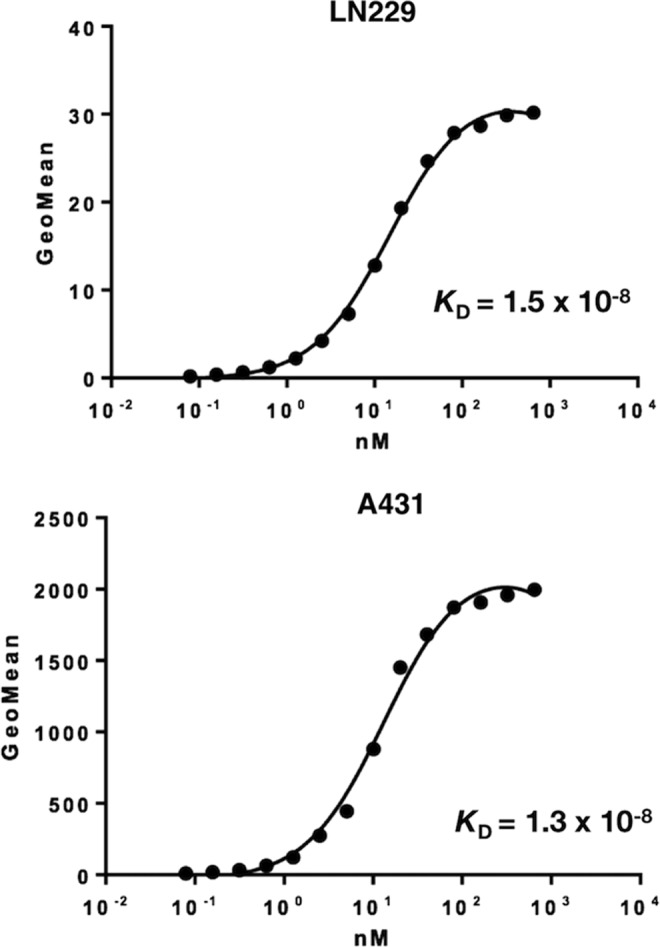
Binding affinity of EMab-51 was determined using flow cytometry. LN229 and A431 cells were suspended in 100 μL of serially diluted EMab-51 (6 ng/mL–50 μg/mL), and secondary anti-mouse IgG was then added. Fluorescence data were collected using a cell analyzer.

We further determined binding affinity of EMab-51 for LN229 and A431 cells using flow cytometry (Fig. 2) and calculated *K*_D_ values for EMab-51 of 1.5 × 10^−8^ M against LN229 or 1.3 × 10^−8^ M against A431, indicating that EMab-51 possesses moderate affinity for EGFR-expressing cell lines.

### Immunohistochemical analysis against oral cancers

**FIG. 3.**
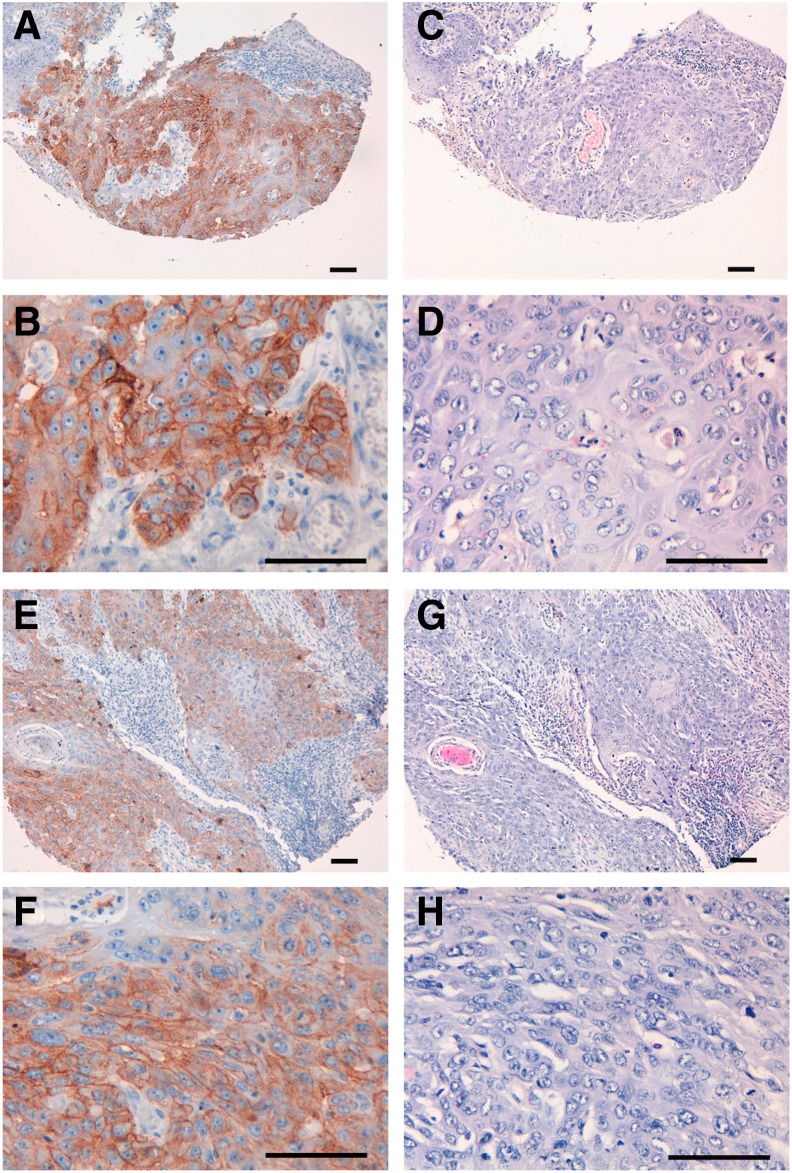
Immunohistochemical analysis by EMab-51 against oral cancers. Sections were incubated with 5 μg/mL of primary EMab-51 for 1 hour at room temperature followed by treatment with EnVision+ Kit for 30 minutes. Color was developed using 3, 3-diaminobenzidine tetrahydrochloride (DAB) for 2 minutes, and sections were then counterstained with hematoxylin. **(A–D)** Case No. 15; **(E–H)** case No. 23; **(A, B, E, F)** immunostaining by EMab-51; **(C, D, G, H)** hematoxylin and eosin staining; scale bar =100 μm.

We investigated the immunohistochemical utility of EMab-51 in human oral cancers because high EGFR expression was observed in oral cancer cell lines, such as HSC-2 and HSC-3 (Fig. 1C). As depicted in Figure 3, EMab-51 stained the membranes of cancer cells of oral squamous cell carcinomas. EMab-51 stained 6 of 38 (15.8%) squamous cell carcinomas in oral cancer tissue array (Supplementary Table S1) and did not stain any other tumor types of oral cancers (Supplementary Table S2).

## Discussion

Establishment of mAbs, which are useful for Western blot, flow cytometry, and immunohistochemical analyses, is often difficult because the design of immunogens and screening methods to develop specific mAbs is different between Western blot and immunohistochemical applications. Furthermore, it is very difficult to produce sensitive and specific mAbs against endogenous proteins. Recently, we developed sensitive and specific antipodocalyxin (PODXL) mAbs using our original technology, and we demonstrated that clone PcMab-47 is useful for flow cytometry, Western blot, and immunohistochemical analyses among anti-PODXL mAbs, warranting consideration of PcMab-47 for investigations of PODXL expression and function in cancers and normal tissues.^(20)^ We further used this technology to generate mAbs that bind to various novel epitopes of PDPN, including LpMab-3,^(21)^ LpMab-7,^(22–24)^ LpMab-10,^(25)^ LpMab-12,^(26)^ LpMab-13,^(27)^ LpMab-17,^(28)^ LpMab-19,^(29)^ and LpMab-21.^(30,31)^ Importantly, these mAbs are useful for Western blot, flow cytometry, and immunohistochemical analyses against PDPN. In this study, we immunized mice with LN229/EGFR or purified recombinant EGFRec from culture supernatants of LN229/EGFRec cells to develop anti-EGFR mAbs, which are useful for Western blot, flow cytometry, and immunohistochemical analyses. Finally, we developed clone EMab-51.

Among 100 anti-EGFR mAbs, 90 clones (90%) were identified as belonging to the IgG_1_ subclass, 5 to the IgG_2a_ subclass, 1 to the IgG_2b_ subclass, and 4 to the IgM class. EMab-51 was also determined to be of the IgG_1_ subclass, precluding confirmation of ADCC or CDC using EMab-51. Thus, in future studies, we will convert the subclass of EMab-51 into mouse IgG_2a_ or IgG_2b_ subclasses or human IgG_1_ and assess their applications for measuring ADCC/CDC activities.^(9,24,32)^

In this study, we used EnVision FLEX Target Retrieval Solution (high pH) for antigen retrieval.^(33–35)^ For immunohistochemical analysis, we typically used citrate buffer (pH 6.0) for antigen retrieval; however, citrate buffer did not improve the signal from EMab-51 use (data not shown).

In conclusion, of 100 clones of anti-EGFR mAbs, EMab-51 was highly efficacious in Western blot analyses and produced strong staining in oral cancers. Hence, EMab-51 could be useful in all present experiments and will likely be an advantageous tool for the pathological identification of EGFR in many cancers.

## Supplementary Material

Supplemental data

Supplemental data

## References

[1] Downward J, Yarden Y, Mayes E, Scrace G, Totty N, Stockwell P, Ullrich A, Schlessinger J, and Waterfield MD: Close similarity of epidermal growth factor receptor and v-erb-B oncogene protein sequences. Nature 1984;307:521–527 6320011 10.1038/307521a0

[2] Ogiso H, Ishitani R, Nureki O, Fukai S, Yamanaka M, Kim JH, Saito K, Sakamoto A, Inoue M, Shirouzu M, and Yokoyama S: Crystal structure of the complex of human epidermal growth factor and receptor extracellular domains. Cell 2002;110:775–787 12297050 10.1016/s0092-8674(02)00963-7

[3] Dokala A, and Thakur SS: Extracellular region of epidermal growth factor receptor: A potential target for anti-EGFR drug discovery. Oncogene 2017;36:2337–2344 27775071 10.1038/onc.2016.393

[4] Mendelsohn J: The epidermal growth factor receptor as a target for cancer therapy. Endocr Relat Cancer 2001;8:3–9 11350723 10.1677/erc.0.0080003

[5] Mendelsohn J, and Baselga J: The EGF receptor family as targets for cancer therapy. Oncogene 2000;19:6550–6565 11426640 10.1038/sj.onc.1204082

[6] Greillier L, Tomasini P, and Barlesi F: Necitumumab for non-small cell lung cancer. Expert Opin Biol Ther 2015;15:1231–1239 26051700 10.1517/14712598.2015.1055243

[7] Uchibori K, Inase N, Araki M, Kamada M, Sato S, Okuno Y, Fujita N, and Katayama R: Brigatinib combined with anti-EGFR antibody overcomes osimertinib resistance in EGFR-mutated non-small-cell lung cancer. Nat Commun 2017;8:14768 28287083 10.1038/ncomms14768 PMC5355811

[8] Kato Y, and Kaneko MK: A cancer-specific monoclonal antibody recognizes the aberrantly glycosylated podoplanin. Sci Rep 2014;4:5924 25080943 10.1038/srep05924 PMC4118152

[9] Kaneko MK, Yamada S, Nakamura T, Abe S, Nishioka Y, Kunita A, Fukayama M, Fujii Y, Ogasawara S, and Kato Y: Antitumor activity of chLpMab-2, a human-mouse chimeric cancer-specific antihuman podoplanin antibody, via antibody-dependent cellular cytotoxicity. Cancer Med 2017;6:768–777 28332312 10.1002/cam4.1049 PMC5387135

[10] Yamada S, Ogasawara S, Kaneko MK, and Kato Y: LpMab-23: A cancer-specific monoclonal antibody against human podoplanin. Monoclon Antib Immunodiagn Immunother 2017;36:72–76 28387591 10.1089/mab.2017.0001

[11] Kaneko MK, Nakamura T, Kunita A, Fukayama M, Abe S, Nishioka Y, Yamada S, Yanaka M, Saidoh N, Yoshida K, Fujii Y, Ogasawara S, and Kato Y: ChLpMab-23: Cancer-specific human–mouse chimeric anti-podoplanin antibody exhibits antitumor activity via antibody-dependent cellular cytotoxicity. Monoclon Antib Immunodiagn Immunother 2017;36:104–112 28504613 10.1089/mab.2017.0014

[12] Fujii Y, Kaneko MK, and Kato Y: MAP tag: A novel tagging system for protein purification and detection. Monoclon Antib Immunodiagn Immunother 2016;35:293–299 27801621 10.1089/mab.2016.0039 PMC5206699

[13] Fujii Y, Kaneko M, Neyazaki M, Nogi T, Kato Y, and Takagi J: PA tag: A versatile protein tagging system using a super high affinity antibody against a dodecapeptide derived from human podoplanin. Protein Expr Purif 2014;95:240–247 24480187 10.1016/j.pep.2014.01.009

[14] Fujii Y, Kaneko MK, Ogasawara S, Yamada S, Yanaka M, Nakamura T, Saidoh N, Yoshida K, Honma R, and Kato Y: Development of RAP tag, a novel tagging system for protein detection and purification. Monoclon Antib Immunodiagn Immunother 2017;36:68–71 28339303 10.1089/mab.2016.0052 PMC5404252

[15] Wang S, Guo P, Wang X, Zhou Q, and Gallo JM: Preclinical pharmacokinetic/pharmacodynamic models of gefitinib and the design of equivalent dosing regimens in EGFR wild-type and mutant tumor models. Mol Cancer Ther 2008;7:407–417 18281523 10.1158/1535-7163.MCT-07-2070

[16] King IC, and Sartorelli AC: The relationship between epidermal growth factor receptors and the terminal differentiation of A431 carcinoma cells. Biochem Biophys Res Commun 1986;140:837–843 3490853 10.1016/0006-291x(86)90710-2

[17] Ghosh MK, Sharma P, Harbor PC, Rahaman SO, and Haque SJ: PI3K-AKT pathway negatively controls EGFR-dependent DNA-binding activity of Stat3 in glioblastoma multiforme cells. Oncogene 2005;24:7290–7300 16007122 10.1038/sj.onc.1208894

[18] Demiroglu-Zergeroglu A, Candemir G, Turhanlar E, Sagir F, and Ayvali N: EGFR-dependent signalling reduced and p38 dependent apoptosis required by gallic acid in malignant mesothelioma cells. Biomed Pharmacother 2016;84:2000–2007 27847212 10.1016/j.biopha.2016.11.005

[19] Reddy KB, Keshamouni VG, and Chen YQ: The level of tyrosine kinase activity regulates the expression of p21/WAF1 in cancer cells. Int J Oncol 1999;15:301–306 10402241 10.3892/ijo.15.2.301

[20] Ogasawara S, Kaneko MK, Yamada S, Honma R, Nakamura T, Saidoh N, Yanaka M, Yoshida K, Fujii Y, and Kato Y: PcMab-47: Novel Antihuman Podocalyxin Monoclonal Antibody for Immunohistochemistry. Monoclon Antib Immunodiagn Immunother 2017;36:50–56 28384052 10.1089/mab.2017.0008 PMC5404275

[21] Oki H, Ogasawara S, Kaneko MK, Takagi M, Yamauchi M, and Kato Y: Characterization of monoclonal antibody LpMab-3 recognizing sialylated glycopeptide of podoplanin. Monoclon Antib Immunodiagn Immunother 2015;34:44–50 25723283 10.1089/mab.2014.0087 PMC4350263

[22] Oki H, Kaneko MK, Ogasawara S, Tsujimoto Y, Liu X, Sugawara M, Takakubo Y, Takagi M, and Kato Y: Characterization of a monoclonal antibody LpMab-7 recognizing non-PLAG domain of podoplanin. Monoclon Antib Immunodiagn Immunother 2015;34:174–180 26090595 10.1089/mab.2014.0090

[23] Kaneko MK, Oki H, Ogasawara S, Takagi M, and Kato Y: Anti-podoplanin monoclonal antibody LpMab-7 detects metastatic legions of osteosarcoma. Monoclon Antib Immunodiagn Immunother 2015;34:154–161 26090592 10.1089/mab.2014.0091

[24] Kato Y, Kunita A, Abe S, Ogasawara S, Fujii Y, Oki H, Fukayama M, Nishioka Y, and Kaneko MK: The chimeric antibody chLpMab-7 targeting human podoplanin suppresses pulmonary metastasis via ADCC and CDC rather than via its neutralizing activity. Oncotarget 2015;6:36003–36018 26416352 10.18632/oncotarget.5339 PMC4742157

[25] Ogasawara S, Oki H, Kaneko MK, Hozumi Y, Liu X, Honma R, Fujii Y, Nakamura T, Goto K, Takagi M, and Kato Y: Development of monoclonal antibody LpMab-10 recognizing non-glycosylated PLAG1/2 domain including Thr34 of human podoplanin. Monoclon Antib Immunodiagn Immunother 2015;34:318–326 26492619 10.1089/mab.2015.0018

[26] Kato Y, Ogasawara S, Oki H, Goichberg P, Honma R, Fujii Y, and Kaneko MK: LpMab-12 established by CasMab technology specifically detects sialylated O-Glycan on Thr52 of platelet aggregation-stimulating domain of human podoplanin. PLoS One 2016;11:e0152912 27031228 10.1371/journal.pone.0152912 PMC4816300

[27] Ogasawara S, Kaneko MK, Honma R, Oki H, Fujii Y, Takagi M, Suzuki H, and Kato Y: Establishment of mouse monoclonal antibody LpMab-13 against human podoplanin. Monoclon Antib Immunodiagn Immunother 2016;35:155–162 27328060 10.1089/mab.2016.0006

[28] Kato Y, Ogasawara S, Oki H, Honma R, Takagi M, Fujii Y, Nakamura T, Saidoh N, Kanno H, Umetsu M, Kamata S, Kubo H, Yamada M, Sawa Y, Morita K, Harada H, Suzuki H, and Kaneko MK: Novel monoclonal antibody LpMab-17 developed by CasMab technology distinguishes human podoplanin from monkey podoplanin. Monoclon Antib Immunodiagn Immunother 2016;35:109–116 26937552 10.1089/mab.2015.0077

[29] Ogasawara S, Kaneko MK, and Kato Y: LpMab-19 recognizes sialylated O-glycan on Thr76 of human podoplanin. Monoclon Antib Immunodiagn Immunother 2016;35:245–253 27564251 10.1089/mab.2016.0031

[30] Kaneko MK, Nakamura T, Honma R, Ogasawara S, Fujii Y, Abe S, Takagi M, Harada H, Suzuki H, Nishioka Y, and Kato Y: Development and characterization of anti-glycopeptide monoclonal antibodies against human podoplanin, using glycan-deficient cell lines generated by CRISPR/Cas9 and TALEN. Cancer Med 2017;6:382–396 28101903 10.1002/cam4.954 PMC5313638

[31] Kato Y, Kunita A, Fukayama M, Abe S, Nishioka Y, Uchida H, Tahara H, Yamada S, Yanaka M, Nakamura T, Saidoh N, Yoshida K, Fujii Y, Honma R, Takagi M, Ogasawara S, Murata T, and Kaneko MK: Antiglycopeptide mouse monoclonal antibody LpMab-21 exerts antitumor activity against human podoplanin through antibody-dependent cellular cytotoxicity and complement-dependent cytotoxicity. Monoclon Antib Immunodiagn Immunother 2017;36:20–24 28234556 10.1089/mab.2016.0045

[32] Yamada S, Kaneko MK, Nakamura T, Ichii O, Konnai S, and Kato Y: Development of mPMab-1, a mouse-rat chimeric antibody against mouse podoplanin. Monoclon Antib Immunodiagn Immunother 2017;36:77–79 28387612 10.1089/mab.2017.0002

[33] Skaland I, Nordhus M, Gudlaugsson E, Klos J, Kjellevold KH, Janssen EA, and Baak JP: Evaluation of 5 different labeled polymer immunohistochemical detection systems. Appl Immunohistochem Mol Morphol 2010;18:90–96 19661787 10.1097/PAI.0b013e3181b0eaad

[34] Sasaki H, Shimizu S, Tani Y, Shitara M, Okuda K, Hikosaka Y, Moriyama S, Yano M, and Fujii Y: Usefulness of immunohistochemistry for the detection of the BRAF V600E mutation in Japanese lung adenocarcinoma. Lung Cancer 2013;82:51–54 23927882 10.1016/j.lungcan.2013.06.014

[35] Sabatasso S, Pomponio C, and Fracasso T: Technical note: EnVision FLEX improves the detectability of depletions of myoglobin and troponin T in forensic cases of myocardial ischemia/infarction. Int J Legal Med 2017 DOI: 10.1007/s00414-017-1575-9 28337600

